# Monitoring the Comparative Safety of SGLT2i vs GLP-1 RA in Older Adults With Type 2 Diabetes by Frailty Status

**DOI:** 10.1093/geroni/igab046.803

**Published:** 2021-12-17

**Authors:** Alexander Kutz, Elisabetta Patorno, Chandrasekar Gopalakrishnan, Dae Kim

**Affiliations:** 1 Brigham and Women's Hospital, Harvard Medical School, Boston, Massachusetts, United States; 2 Brigham and Women's Hospital and Harvard Medical School, Boston, Massachusetts, United States; 3 Hebrew SeniorLife, Boston, Massachusetts, United States

## Abstract

Using Medicare (4/2013-12/2017), we conducted 9 sequential analyses of patients with type 2 diabetes initiating SGLT2i vs. GLP1-RA mimicking the accrual of new data every 6 months to monitor SGLT2i safety with respect to diabetic ketoacidosis (DKA) since their U.S. approval. For each analysis, we estimated cumulative HRs (95% CIs) after 1:1 propensity score matching on >70 covariates comparing treatments within frail and non-frail patients. By analysis 1, SGLT2i were associated with a higher DKA rate vs. GLP-1RA in both frail and non-frail patients, but results were highly imprecise due to few events. With the accrual of more DKA events, precision of the estimates continued to improve through analysis 9 [HR=2.95 (95% CI, 1.19-7.31)] in frail patients; [HR=1.77 (1.15, 2.75)] in non-frail patients], with sufficiently precise estimates by analysis 6 in frail patients [HR=2.80 (95% CI, 1.03, 7.61)] and by analysis 7 in non-frail patients [HR=1.62 (95% CI, 1.01, 2.57)].

